# Bridging Metabolic-Associated Steatotic Liver Disease and Cardiovascular Risk: A Potential Role for Ketogenesis

**DOI:** 10.3390/biomedicines12030692

**Published:** 2024-03-20

**Authors:** Rafael Suárez del Villar-Carrero, Agustín Blanco, Lidia Daimiel Ruiz, Maria J. García-Blanco, Ramón Costa Segovia, Rocío García de la Garza, Diego Martínez-Urbistondo

**Affiliations:** 1 Grupo de Riesgo Vascular, Sociedad Española de Medicina Interna (SEMI), 28016 Madrid, Spainmariajgarciablanco@gmail.com (M.J.G.-B.); rgdelagarza@unav.es (R.G.d.l.G.); 2 Grupo de Trabajo Prevención Secundaria y Alto Riesgo Vascular, Sociedad Española Arteriosclersosis (SEA), 08029 Barcelona, Spain; 3 Servicio de Urgencias, Hospital Universitario HM Monteprincipe, 28660 Madrid, Spain; 4 Unidad de Riesgo Cardiovascular, Departamento de Medicina Interna, Hospital Universitario 12 de Octubre, 28041 Madrid, Spain; 5 Grupo de Estudio de Nutrigenómica y Nutrición Personalizada, IMDEA Alimentación, 28049 Madrid, Spain; lidia.daimiel@alimentacion.imdea.org; 6 CIBEROBN, Instituto Carlos III, 28029 Madrid, Spain; 7 Servicio de Medicina Interna, Hospital Central de la Defensa Gómez Ulla, 28047 Madrid, Spain; 8 Facultad de Medicina, Universidad de Alcalá, 28801 Alcalá de Henares (Madrid), Spain; 9 Área de Medicina Vascular, Departamento de Medicina Interna, Clínica Universidad de Navarra, 28027 Madrid, Spain

**Keywords:** cardiovascular risk, cardiovascular disease, ketone bodies, ketogenesis, MASLD

## Abstract

The prevalence of cardiovascular diseases (CVDs) is a growing global health concern. Recent advances have demonstrated significant reductions in acute cardiovascular events through the management of modifiable cardiovascular risk factors. However, these factors are responsible for about 50% of the global cardiovascular disease burden. Considering that CVDs are one of the top mortality causes worldwide, the concept of residual cardiovascular risk is an important emerging area of study. Different factors have been proposed as sources of residual risk markers, including non-HDL particles characterization, as well as inflammation measured by serum and imaging technics. Among these, metabolic-associated steatotic liver disease (MASLD) remains controversial. Two opposing viewpoints contend: one positing that fatty liver disease merely reflects classical risk factors and thus adds no additional risk and another asserting that fatty liver disease independently impacts cardiovascular disease incidence. To address this dilemma, one hypothetical approach is to identify specific hepatic energy-yielding mechanisms and assess their impact on the cardiovascular system. Ketogenesis, a metabolic intermediate process particularly linked to energy homeostasis during fasting, might help to link these concepts. Ketogenic metabolism has been shown to vary through MASLD progression. Additionally, newer evidence supports the significance of circulating ketone bodies in cardiovascular risk prediction. Furthermore, ketogenic metabolism modification seems to have a therapeutic impact on cardiovascular and endothelial damage. Describing the relationship, if any, between steatotic liver disease and cardiovascular disease development through ketogenesis impairment might help to clarify MASLD’s role in cardiovascular risk. Furthermore, this evidence might help to solve the controversy surrounding liver steatosis impact in CVD and might lead to a more accurate risk assessment and therapeutic targets in the pursuit of precision medicine.

## 1. Introduction

Cardiovascular diseases (CVD) represent a major concern in preventive health. An estimated 17.9 million people died from CVDs in 2019, representing 32% of global deaths worldwide. Of these deaths, 85% were due to heart attack and stroke. In the United States, CVD remains the leading cause of death for both men and women, accounting for approximately one in every four deaths [1]. According to the American Heart Association, in 2020, nearly half of U.S. adults (48%) were estimated to have some form of CVD, with the prevalence slightly higher in men (50.5%) than in women (47.3%) [2]. In Europe, the burden of CVD is similarly worrying, with cardiovascular diseases being the leading cause of death, accounting for 45% of all deaths in Europe and 37% in the European Union. The prevalence of CVD in European adults is estimated to be around 49% in men and 38% in women [3].

The prevention of CVD is a critical aspect of public health strategies, providing a Global action plan for the prevention and control of non-communicable diseases (NCD). This plan aims to reduce the number of premature deaths from NCDs by 25% by 2025 through different global targets [4]. Lifestyle modifications, such as adopting a balanced diet, engaging in regular physical activity, avoiding nicotine exposure, and maintaining a healthy weight, play a pivotal role in reducing the risk of CVD. These measures address key modifiable risk factors, including hypertension, dyslipidemia, diabetes, and obesity, which are known to significantly increase the likelihood of developing CVD. Together, the control of hypertension, dyslipidemia, dysglucemia, sleep care, physical activity performance, taking care about nutrition, avoiding tobacco, and maintaining a healthy weight are known as life’s essential eight [5]. Investigations of the pathogenesis of CVD have identified several potential pathways involving inflammation, endothelial function, atherosclerosis, cardiac stress and remodeling, hemostatic factors, microbiota, and epigenetics, among others [6,7,8,9,10].

At present, classic cardiovascular risk factors can only explain approximately 57% of cardiovascular events in women and 52% in men, accounting for a 10-year all-cause mortality of 22.2% and 19.1%, respectively [11]. This limitation highlights the growing importance of clinical and translational research in studying residual risk due to other characteristics. In this context, non-HDL molecules and the triglyceride content of cholesterol-carrying particles have demonstrated a significant and likely causal role in cardiovascular risk. Non-HDL cholesterol includes all atherogenic lipoproteins and is considered a better marker of risk than LDL cholesterol alone [12,13,14]. Elevated levels of non-HDL cholesterol are associated with an increased risk of atherosclerotic cardiovascular disease. Lipoprotein (a), or Lp (a), is another lipid-related risk factor receiving increased attention. Lp (a) is a unique lipoprotein particle with a structure similar to LDL cholesterol, but with an additional protein called apolipoprotein (a). Elevated levels of Lp (a) have been independently associated with increased risk of cardiovascular diseases, including coronary heart disease and stroke [15]. Lp (a) is considered a genetically determined risk factor, with concentrations largely unaffected by lifestyle changes or most lipid-lowering medications [16,17].

Local and systemic inflammation plays a significant role in the pathogenesis of CVD [18,19,20,21,22,23,24,25,26]. Hs-CRP has been widely recognized as a marker of systemic inflammation and an independent predictor of cardiovascular events. Furthermore, IL-1 and IL-6 are key cytokines involved in inflammatory processes and have been linked to atherosclerosis progression [19,20,21]. These findings underscore the need for a broader approach to cardiovascular risk assessment and management, encompassing both traditional and emerging risk factors. Identifying and targeting these residual risks could lead to more effective strategies for preventing cardiovascular diseases.

## 2. Liver Steatotic Disease as a Potential Residual Cardiovascular Risk Factor

The progressive increase in the global prevalence of hepatic steatosis disease raises the question of whether this condition might play a role in predicting residual cardiovascular risk [27]. Steatotic liver disease (SLD), previously known as non-alcoholic fatty liver disease (NAFLD), has become the most common cause of liver disease in developed countries today, as up to 30–40% of the global population may be affected by NAFLD [28,29]. This incidence and persistence of simple steatosis in patients is associated with the development of more advanced forms of the disease with a higher morbimortality, such as non-alcoholic steatohepatitis (NASH), cirrhosis, or the development of hepatocellular carcinoma [29]. Furthermore, the increasing prevalence of this condition among young individuals confers additional risk due to the prolonged duration of disease exposure [29]. However, the number of deaths directly attributable to liver disease itself is relatively limited [29].

**Figure 1:**
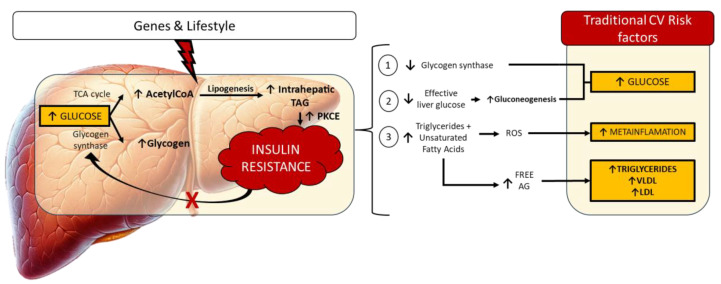
Role of simplified MASLD pathogenesis in classic cardiovascular risk factor development. TCA: Tricarboxylic acid; TAG: Triglycerides; ROS: Reactive oxygen species; AG: Acylglycerides; CV: Cardiovascular. Upwards arrows: increases; downwards arrows: decreases; red cross: blocks; red lighting symbol: interferes or impacts. Legend: Both pernicious genes and lifestyle have a role in the incremental concentration of glucose in the liver, which is metabolized through the TCA cycle and the glucogen synthesis pathways, among others. An excess in Acetyl CoA provides a higher concentration of intrahepatic acyl glycerides, which is directly linked to insulin resistance, providing reduced glycogen synthesis, a need for higher levels of insulin to provide glucose for energetic purposes (effective liver glucose), which activates intrahepatic gluconeogenesis and an excess in triglycerides, leading to a less efficient metabolization, with Radical Oxygen Species production and a higher plasma concentration of triglycerides and cholesterol particles contributing to a higher cardiovacular risk through classical risk factors [30,31,32,33].

Interestingly, steatotic liver disease is deeply linked to metabolic and cardiovascular disease [30]. Insulin resistance, a hallmark of metabolic syndrome, plays a central role in the development of NAFLD by promoting the accumulation of fat in the liver and exacerbating liver inflammation and damage [31,32,33]. Liver steatosis also contributes to alterations in lipid metabolism and glucose regulation (Figure 1) [33]. As research has advanced, the association between NAFLD and classic cardiovascular risk factors has become more evident, including hypertension, dyslipidemia, obesity, and type 2 diabetes mellitus [34]. Thus, the conceptualization of steatotic liver disease (SLD) has significantly progressed, with a particular focus on distinguishing between metabolic and alcohol-related factors while avoiding stigmatizing terms for patients. The term non-alcoholic fatty liver disease (NAFLD) has been re-evaluated due to concerns that it might overlook the nuances of alcohol’s role in liver steatosis [35]. Acknowledging that both excessive and moderate alcohol consumption can influence liver health, a more refined diagnostic approach has been advocated. This shift to the broader category of SLD allows for a more inclusive understanding, taking into account varying levels of alcohol intake. The new nomenclature distinguishes between excessive (Metabolic and Alcohol Steatotic Liver Disease—MetALD) and moderate or no alcohol intake (Metabolic-Associated Steatotic Liver Disease—MASLD) [35]. Although the repercussions on the disease epidemiology are still under evaluation, with some data pointing to a low reclassification capacity of the new definition [36], the inclusion of a metabolic disturbance basis on SLD categorization reaffirms a new paradigm in the comprehension of this systemic disease. However, the question remains open: Does MASLD contribute independently to cardiovascular residual risk beyond traditional factors?

Cardiovascular events are the most frequent cause of morbimortality in patients with NAFLD [29]. Consequently, interest in studying the potential causal relationship between SLD and cardiovascular disease has surged [27,37,38]. Initially, some epidemiological assessments pointed out that hepatic lipid accumulation mirrors an individual’s metabolic milieu but does not represent an independent risk factor for CV events [39,40]. This concept is also included in the current European Society of Cardiology guidelines for cardiovascular risk prevention [41]. In these cohorts, although some effects of steatosis and liver fibrosis could be addressed, this prediction capacity was lost when further adjustments were applied. However, other investigations suggest that NAFLD might proffer an additional, independent prognostic value for cardiovascular events, including a biopsy-based NAFLD stage correlation with the probability of cardiovascular arrest [42] and a mendelian randomization study, which found an independent link between NAFLD and CVD when gene function adjustments were included [43]. Nevertheless, biopsy or genetic studies are too risky, expensive, and complicated to be efficient in the risk factor scenario [44]. Thus, hepatic steatosis may be considered a surrogate metric for metabolic risk when evaluated through non-invasive techniques such as ultrasound, MRI, and transient elastography [45,46] and even validated serologic indices [47]. Several epidemiological studies have explored the association between non-invasively assessed SLD and metabolic and cardiovascular risk regarding quality of life [48], as well as the impact of lifestyle modification [49], incidence of T2DM [50], and incidence of cardiovascular events [51]. Various prospective cohort analyses and meta-analyses, grounded in liver biopsy results [42] and non-invasive biomarkers [52,53], have buttressed this hypothesis and precipitated a specific AHA statement on this subject [27] (Table 1).

Although further meticulous epidemiological research should be performed in the field, the pursuit of specific hepatic metabolic pathways linking MASLD to CVD is settled. In this context, the production of ketone bodies through the Randle cycle presents several interesting features: (i) liver exclusiveness, (ii) ketone synthesis variation along the MASLD spectrum [75], and (iii) potential prediction capacity in cardiovascular disease [76]. Ketogenesis predominantly occurs in the liver, where fatty acids are converted into ketone bodies, namely acetoacetate, beta-hydroxybutyrate, and acetone. Ketone bodies exhibit biphasic alterations in MASLD patients, escalating during initial disease phases and waning during advanced stages [75]. Ketone bodies have been related to CVD in two ways. On the one hand, their reducing effect on oxidative stress and inflammation was suggested to protect from CVD [77,78]; on the other hand, they were found to be associated with cardiovascular risk in CVD-naïve patients [76]. Thus, further elucidation of the role of ketogenesis may contribute to the understanding of the link between SLD and cardiovascular risk. Thus, assessing ketogenesis may contribute to the understanding of the link between SLD and cardiovascular risk. 

## 3. Metabolic Associated Steatotic Liver Disease Role in Ketogenesis Impairment

**Figure 2:**
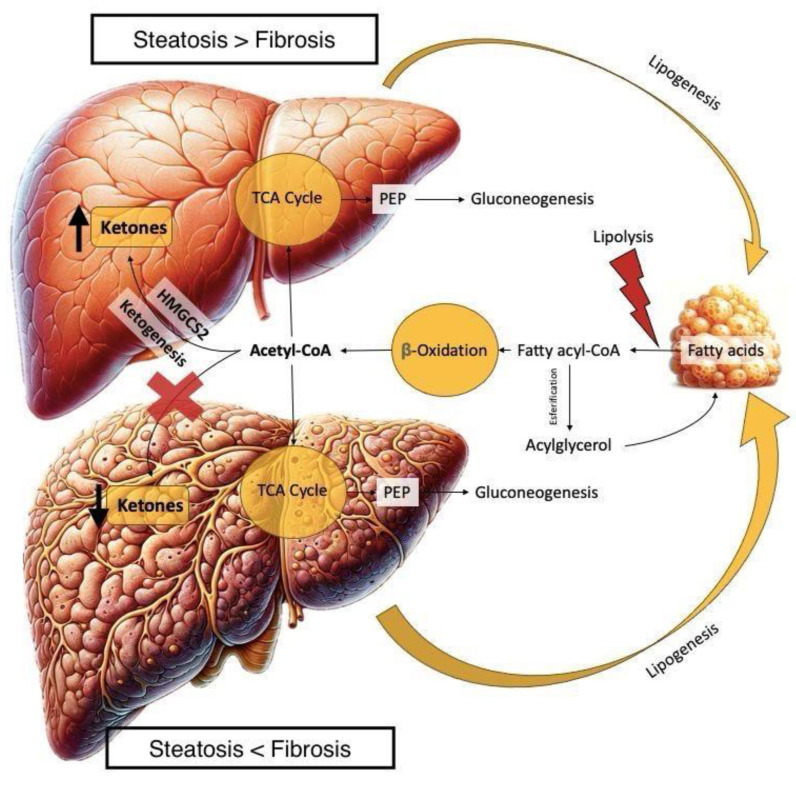
TCA: Tricarboxylic acid; PEP: Phosphonyl pyruvate. Legend: A dual result of triglyceride exposure might be expected depending on the MASLD status, as a higher exposure to beta-oxidation products leads to a higher production of ketone bodies in the first stages of SLD, while, as fibrosis progresses, ketone metabolism is reduced as a potential early marker of liver metabolic dysfunction [75]. Upward arrows indicate an increase in the synthesis of ketone bodies, activation of the TCA cycle, and gluconeogenesis. Downward arrows would represent a decrease in the processes of lipogenesis and the accumulation of fatty acids. The red cross would indicate the inhibition of the hydroxy-methyl-glutaril-CoA (HMG-CoA) complex pathway, thus decreasing the production of ketone bodies. The red lightning symbol suggests stress or damage leading to steatosis and possibly hepatic fibrosis.

Ketogenesis is initiated in the mitochondrial matrix of hepatocytes and entails a cascade of enzymatic reactions, catalyzed by lipases, enzymes of betaoxidation, and HMG-CoA synthase [79]. The generated ketone bodies acetoacetate, betahydroxybutyrate (BHB) and acetone can serve as energy source, particularly furnishing essential energy to the brain and the cardiovascular system during periods of fasting or carbohydrate restriction [80]. The influence of MASLD on ketogenesis is bi-phasic. Initially, ketone body production is augmented due to an increase in beta-oxidation while other hepatic features remain clinically normal [81]. Subsequently, as steatohepatitis and fibrosis unfold, production is reduced due to a decrease in HMG-CoA activity [80,82] (Figure 2).

### 3.1. Ketogenesis Impairment in MASLD

In the early stage of MASLD, hepatic lipogenesis is increased, resulting in liver steatosis. This is associated with increased fatty acid uptake and synthesis [83] and insufficient fatty acid conversion to ketone bodies [84]. This impaired ketogenesis could deleteriously impact mitochondrial functionality, given the intimate relationship between the ketogenesis pathway and mitochondrial integrity [85]. The accumulation of fatty acids within hepatocytes can provoke mitochondrial dysfunction and oxidative stress, igniting a vicious cycle that exacerbates hepatic lipid accumulation, induces hepatic injury, and propels inflammation and fibrosis, favoring the progression from simple steatosis to metabolic-associated steatohepatitis (MASH) [86]. Ketogenesis can be influenced by multiple factors, including insulin resistance, nutrient availability, and signaling cascades across various cellular receptors and pathways. Insulin resistance, a pivotal actor in SLD onset and progression, notably exerts a suppressive impact upon ketogenesis [87]. Additionally, the peroxisome proliferator-activated receptor-alpha (PPARα), instrumental in regulating fatty acid metabolism, becomes substantively involved in the regulatory mechanisms of this process comprising hepatic fatty acid and plasma lipoprotein metabolism during nutritional transition and the regulation of hepatic inflammatory response [88].

**Figure 3:**
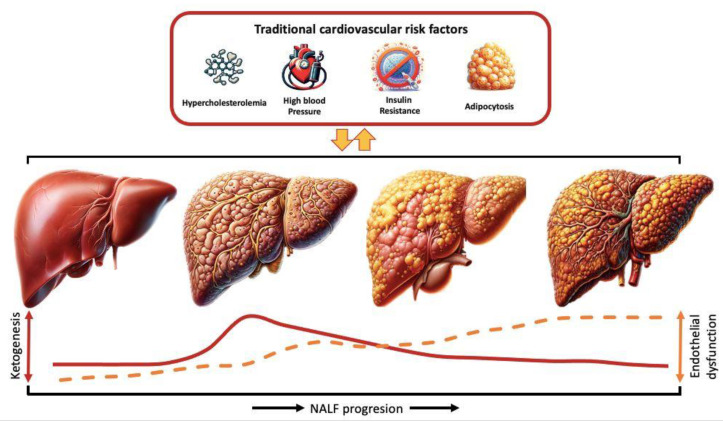
Potential interrelationship between traditional cardiovascular risk factors and SLD in the progression of endothelial dysfunction and ketone body production. Legend: The red line describes the evolution of ketone body production through NAFL evolution, while the yellow line describes the evolution of endothelial dysfunction [42,77].

When NAFLD advances to NASH, the interplay between impaired ketogenesis and hepatic lipid metabolism diversifies, adding inflammation and cellular injury to lipid accumulation. In this context, impaired ketogenesis may intensify hepatic lipid accumulation, oxidative stress, and mitochondrial dysfunction, thereby contributing to hepatocellular injury and inflammation [75]. Then, as the pathophysiological process evolves through the progression toward fibrosis and cirrhosis, ketone production is diminished. This metabolic disturbance might affect systemic energy homeostasis especially in the brain and heart [79], which are the main targets of CVD. Thus, circulating ketone bodies might serve as indirect hepatic function markers with specific cardiovascular implications, but these should be completely understood, compelling the interaction between SLD progression, ketone body production, and endothelial dysfunction (Figure 3).

### 3.2. Ketogenesis Impairment in Cardiovascular Disease

Beta-hydroxybutyrate is the most abundant in plasma, constituting more than 90% of the total ketone bodies, though acetoacetate and acetone also occur in blood samples [80]. Particularly during glucose-limiting conditions such as prolonged fasting or rigorous physical exertion, these ketone bodies are indispensable, ensuring energy provision predominantly for the cerebrum and myocardium [80]. The affinity of these fuel transfer metabolites for the major organs involved in cardiovascular events is still intriguing. Emerging evidence underscores the advantageous impact of ketone bodies on cardiac metabolic adaptability under pathological scenarios like heart failure (HF) [89,90,91,92]. Hence, during energy deficits, the malfunctioning heart may escalate ketone utilization, acting as a compensatory response to its amplified energy demands [91,92]. Yet the question remains: does reduced ketogenesis improve or deteriorate CVD prognosis?

Preliminary findings suggest that ketone synthesis in apparently healthy individuals is associated with higher cardiovascular risk. Inadequate ketone body synthesis might aggravate cardiac malfunction by depriving the heart muscle of an essential energy source during metabolic distress periods [91,92]. A similar theory has been advanced concerning arteriosclerosis [93].

Conversely, dysregulated ketone metabolism and heightened BHB concentrations, frequently observed in diabetic cohorts, might elevate cardiovascular risk by fostering oxidative stress and endothelial malfunction [94]. It appears that maintaining equilibrium in ketone metabolism, evading both deficiency and surplus, is imperative for cardiovascular integrity [95]. Some investigations, specifically from the MESA cohort, have appraised the nexus between circulating ketone bodies and CVD occurrence. Preliminary results show that augmented endogenous ketone body levels correlate with a higher incidence and mortality rate of CVD among populations without prior events due to CVD. This potentially positions ketone bodies as prospective biomarkers for cardiovascular risk evaluation [76]. 

Thus, the interpretation of ketone bodies in the clinical setting remains unclear. On the one hand, further ketone synthesis in apparently healthy individuals leads to higher cardiovascular risk, while on the other hand, ketone body insufficiency in diseased patients seems to be associated with further morbidity and mortality. Hence, different hypotheses might arise from this controversy: Are ketone bodies an early biomarker of cardiovascular disease? This approach might mean that people generating a relative excess of ketones while being apparently healthy are adapting to a thinner metabolic health equilibrium through flexible homeostatic resources such as ketone bodies. However, an increased ketogenesis could be a consequence of other metabolic disturbances such as SLD progression, providing further cardiovascular and metabolic risk through other pathways such as dyslipidemia or serum glucose control, but then, how is mild ketone body increase associated with a better prognosis in secondary cardiovascular prevention? Although this fact could be elucidated by a higher capacity of these patients to provide energetic balance through accessory pathways, this would not correlate with the apparent harmful effect of liver steatosis progression, which is linked to a higher production of ketones during the intermediate phase of MASH. In addition to its role in energy supply, BHB operates as an epigenetic regulator, curbing histone deacetylases and subsequently modifying gene transcription [96]. This modulatory capability suggests that ketogenesis perturbations altering BHB concentrations could potentially impact cardiac gene expression, introducing another layer of complexity to cardiovascular pathophysiology. Therefore, an in-depth exploration of the relationships between hepatic ketogenesis and cardiovascular health is key to uncovering clinically significant ties between SLD and CVD.

### 3.3. Impaired Ketogenesis: Bridging Non-Alcoholic Fatty Liver Disease (NAFLD) and Cardiovascular Disease (CVD)

Mediation analyses to prove the connection between SLD and CVD through circulating ketone bodies have been appraised. Post A et al. investigated the association between NAFLD and circulating ketone bodies in a cohort of 6297 participants and determined the extent to which NAFLD and circulating ketone bodies were associated with all-cause mortality [97]. An elevated FLI as a marker of liver steatosis was independently associated with an increased risk of mortality. Higher total ketone bodies were also associated with an increased mortality risk. Mediation analysis suggested that the association of elevated FLI with all-cause mortality was in part mediated by ketone bodies (proportion mediated: 10%, *p* < 0.001).

This association might be mechanistically rooted in impaired lipid metabolism, which fosters dyslipidemia, typified by an augmentation of triglycerides and low-density lipoprotein cholesterol (LDL-C), and a decrease in high-density lipoprotein cholesterol (HDL-C) levels [98]. Furthermore, the intricate web intertwining NAFLD and CVD might be influenced by inflammatory pathways, especially in the context of the more aggressive non-alcoholic steatohepatitis (NASH). This latter condition is associated with systemic inflammation and a surge in pro-inflammatory cytokines such as tumor necrosis factor-alpha (TNF-α) and interleukin-6 (IL-6), which are pivotal in atherosclerosis and CVD pathogenesis [99,100]. Moreover, the potential role of impaired ketogenesis in modulating nitric oxide (NO) bioavailability and, consequently, impacting endothelial function, which is pivotal for vascular well-being, cannot be ignored. A decrease in endothelial NO production is intrinsically linked with endothelial dysfunction, a harbinger of atherosclerosis and subsequent CVD [101].

Intriguingly, the nuanced effect of impaired ketogenesis is somewhat illuminated by the impact of externally provided ketone production on a myriad of markers like dyslipemia [102,103], dysglycemia [104,105,106], appetite and obesity [107], blood pressure [108], meta inflammation [78,109], and endothelial dysfunction [77,110], providing regulation of insulin and glucagon role in beta-oxidation, glycolysis, gluconeogenesis, and NO synthase in the hemodynamic control of vascular system. These findings, as well as the predictive value of circulating ketone bodies for CVD, point to an independent effect of impaired ketogenesis on CVD and a potential benefit of circulating ketone bodies for assessing cardiovascular risk (Figure 3).

Some novel therapeutic tools to treat metabolic disturbances, such as sodium-glucose co-transporter 2 inhibitors (SGLT2i), might also help to elucidate the consequential impact of ketogenesis in CVD. By obstructing glucose reabsorption in proximal renal tubules and by amplifying urinary glucose excretion, SGLT2i not only mitigates hyperglycemia but also reduces cardiovascular events and mortality in diabetic patients with close monitoring to avoid euglycemic diabetic ketoacidosis [111,112,113]. Furthermore, SGLT2i might augment glucagon secretion [114]. On a contrasting note, SGLT2i, while inducing a mild ketosis, paves the way for several metabolic adaptations, potentially conferring assorted benefits. For example, mild ketosis, propelled by an elevation in circulating ketone bodies like BHB, could offer an alternative energy substrate for various tissues when glucose utilization is jeopardized [115]. BHB also plays roles in signaling activities, potentially exerting anti-inflammatory and anti-aging effects by inhibiting the NLRP3 inflammasome and reducing oxidative stress [86,116,117,118]. For patients with type 2 diabetes, the utilization of ketone bodies as an alternate fuel might aid in safeguarding cardiac and renal functions. These benefits have translated into a potential role of ketogenesis in SGLT2i protection against heart failure, decompensation, and kidney failure [116,117,118].

The clinical approach to cardiovascular disease represents na urgent for the research of new pathways that may elucidate the connection between different pathogenic mechanisms, deriving causal biomarkers and potential therapeutic targets. The finding of new pathways may help to finally control the cardiovascular pandemic. Thus, the available pathophysiological and epidemiological data hint at the impact of impaired ketogenesis as a potential mediator between SLD and cardiovascular disease. This statement could be set based on liver exclusiveness in ketone production, the fluctuations in ketogenesis according to MASLD status, and evidence for the role of ketone bodies in cardiovascular disease. These concepts might pinpoint impaired ketogenesis as a distinct biological mechanism linking MASLD with CVD. For these reasons, the authors consider that future research should support the effort to develop and perform specific studies to furnish mechanistic evidence of this connection. Subsequent evidence may aid in refining our understanding of residual cardiovascular risk, providing further knowledge of both processes, and thereby enhancing diagnostic and therapeutic precision and fostering the progress of precision medicine.

## Figures and Tables

**Table 1:** Studies providing evidence on NAFLD invasive and non-invasive assessment in the prediction of cardiovascular disease [27].

Diagnostic NAFLD	Reference	Patients, *n*	Type of Study	Impact of the NAFLD
**Ultrasound**				
	Stepanova and Younossi, 2012 [54]	20,050	Prospective	OR, 1.23 for CVD events
	Haring et al., 2009 [55]	4160	Prospective	HR, 6.22 for all-cause mortality and CVD
	Kim et al., 2012 [56]	4023	Cross-sectional	OR, 1.32 for CAC > 10
	Targher et al., 2007 [57]	2839	Cross-sectional	OR, 1.49 for DKA DBP, and cerebrovascular disease in type 2 DM
	Tsutsumi T et al., 2021 [58]	2306	Prospective	HR, 1.08 independently with worsening CVD
	Hamaguchi et al., 2007 [59]	1637	Prospective	HR, 4.1 for nonfatal CVD events
	Yoshitaka and al, 2017 [60]	1647	Prospective	HR, 10.4 not overweight, 3.1 overweight for incident CV events
	Wong et al., 2011 [61]	612	Prospective	OR, 2.31 for significant coronary artery disease (>50% obstruction)
	Santos et al., 2007 [62]	505	Cross-sectional	OR, 1.73 for coronary calcification
	Mantovani et al., 2016 [63]	286	Retrospective	OR, 6.73 for incident cardiovascular events in type 1 diabetes
**CT**				
	Mahfood Hadad et al., 2016 [64]	25,837 (11 studies)	Meta-analysis	RR, 1.77 for incident CVD, 1.43 for cardiovascular mortality
	Zhou et al., 2018 [65]	8346 (6 studies)	Meta-analysis	OR, 2.20 for incident CVD in patients with diabetes
	Mellinger et al., 2015 [66]	3014	Cross-sectional	OR, 1.20 for CAC score >90th percentile for age
	Assy et al., 2010 [67]	61	Cross-sectional	OR, 2.03 for coronary calcification
**Ultrasound/CT**				
	Chen et al., 2010 [68]	295	Cross-sectional	OR, 2.46 for CAC > 100
**Liver biopsy**				
	Simon et al., 2022 [42]	422	Prospective	HR, 2.15 for MACE
	Ji Hye Park et al., 2021 [69]	398	Cross-sectional	OR, 4.07 increased risk of ASCVD for NASH OR, 8.11 increased risk of ASCVD for advanced fibrosis
	Ekstedt et al., 2015 [70]	229	Retrospective	HR, 1.55 for CVD mortality
**Fatty Liver Index**				
	Chun HS et al., 2023 [71]	78,762	Cross-sectional	OR, 1.10 for CVD history in MAFLD OR, 1.40 for high probability of ASCVD in MAFLD OR, 1.22 for high probability of ASCVD in NAFLD
	Pais et al., 2016 [72]	5671	Retrospective	The severity of NAFLD correlates with CIMT and the severity of carotid plaque
	Lee J et al., 2020 [73]	1173	Prospective	OR, 1.70 for CAC progression in patients with NAFLD
	Pennisi et al., 2021 [74]	542	Cross-sectional	OR, 1.62 risk factors for ASCVD in patients with steatosis OR, 1.67 risk factors for ASCVD in patients with severe fibrosis

NAFLD: non-alcoholic fatty liver disease; OR: Odds ratio; CVD: cardiovascular disease; HR: hazard ratio; CAC: coronary artery calcium; DKA: diabetic ketoacidosis; DBP: diastolic blood pressure; RR: relative risk; MACE: major adverse cardiovascular event; ASCVD: atherosclerotic cardiovascular disease; NASH: non-alcoholic steatohepatitis; MAFLD: metabolic dysfunction-associated fatty liver disease.
